# Butterfly hematoma after traumatic intercourse

**DOI:** 10.11604/pamj.2015.20.317.6660

**Published:** 2015-04-01

**Authors:** Fouad Hajji, Ahmed Ameur

**Affiliations:** 1 Department of Urology, Mohammed V Military University Hospital, Rabat, Morocco

**Keywords:** Butterfly hematoma, traumatic intercourse, penis

## Image in medicine

**Figure 1:**
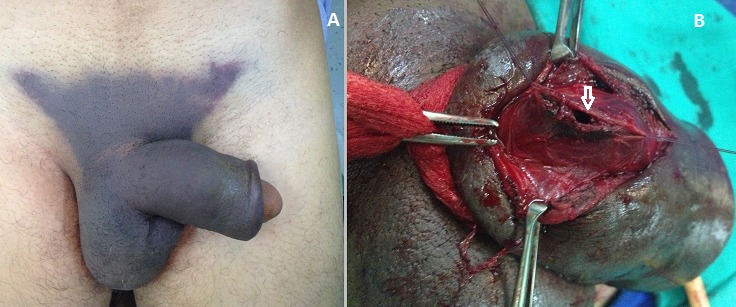
A) penis with a butterfly hematoma in the lower abdominal wall; B) tear of tunica albuginea at the right corpus cavernosum (confirmed by surgical exploration)

A 33-year-old man presented with penile pain, swelling and ecchymosis after striking his erect penis against his partner's perineum during intercourse. At the moment of injury, he recalled hearing a cracking sound with tearing sensation, followed by instant penile pain and immediate loss of erection. Physical examination revealed a flaccid, swollen and ecchymotic circumcised penis with a butterfly hematoma in the lower abdominal wall, scrotum and perineum (A). The glans appeared normal and the testicles felt structurally normal. However, the phallus was deviated to the left side and the penile shaft had a palpable defect on its right base. This examination suggested rupture of the corpus cavernosum of the penis with hematoma extravasation outside Buck's fascia. In a patient presenting with a butterfly hematoma after traumatic intercourse, differential diagnosis should also include rupture of deep dorsal penile vessels with hematoma extravasation outside Buck's fascia, rupture of superficial dorsal penile vein or non-specific dartos bleeding. The patient denied any voiding difficulties or gross haematuria, and had neither blood at the meatus nor palpable bladder. Moreover, there was no microscopic hematuria on urinalysis. Surgical penile exploration was performed through a right-sided incision and confirmed the tear of tunica albuginea at the right corpus cavernosum (B, arrow). Rupture of the corpus cavernosum of the penis or penile fracture is an uncommon condition, but is reported to occur more frequently during intercourse than avulsion of dorsal penile vessels. It can be accompanied by urethral injury in 10 to 20% of cases. Our patient had neither disrupted vasculature nor associated urethral injury, and both tunica albuginea and Buck's fascia lacerations were repaired with an uneventful postoperative course.(Figure 1)

